# ^18^F-Labelled Intermediates for Radiosynthesis by Modular Build-Up Reactions: Newer Developments

**DOI:** 10.1155/2014/812973

**Published:** 2014-06-23

**Authors:** Johannes Ermert

**Affiliations:** Institut für Neurowissenschaften und Medizin, INM-5: Nuklearchemie, Forschungszentrum Jülich, 52425 Jülich, Germany

## Abstract

This brief review gives an overview of newer developments in ^18^F-chemistry with the focus on small ^18^F-labelled molecules as intermediates for modular build-up syntheses. The short half-life (<2 h) of the radionuclide requires efficient syntheses of these intermediates considering that multistep syntheses are often time consuming and characterized by a loss of yield in each reaction step. Recent examples of improved synthesis of ^18^F-labelled intermediates show new possibilities for no-carrier-added ring-fluorinated arenes, novel intermediates for tri[^18^F]fluoromethylation reactions, and ^18^F-fluorovinylation methods.

## 1. Introduction

The positron emitter fluorine-18 is a commonly used radionuclide in molecular imaging with positron emission tomography (PET) due to its advantageous nuclear properties. Thus, it finds wide application as noninvasive, quantitative, and versatile modality in medical diagnosis, research, and drug development [1]. Fluorine-18 has a short half-life of 109.7 min which only allows time-limited syntheses and study protocols. The methods for introducing this short-lived radionuclide into organic molecules thus require fast chemistry, and it is desirable to introduce the ^18^F-label during the last possible synthetic step.

A further aspect is the stoichiometry of ^18^F-chemistry that differs from “cold” fluorinations. The radionuclide is produced in low (nano- to picomolar) amounts and its concentration in reaction mixtures is several orders of magnitude lower than the precursor concentration. Furthermore, the syntheses of the radiotracers have to be performed in closed, lead-shielded hot cells, which necessitates an easily applicable and remote-controlled process. Thus, besides the development of more efficient and flexible ^18^F-labelling methods new technological approaches have been examined, especially in the field of microfluidic chemistry [2–10]. The development of a reliable ^18^F-labelling technique together with an automatic synthesis module is a major prerequisite of routine production of ^18^F-labelled PET radiopharmaceuticals [11–13].

Methods for the introduction of [^18^F]fluorine into organic molecules can be divided into two groups, namely, direct and indirect. The direct method entails introduction of [^18^F]fluorine without changing the carbon skeleton structure of the molecule. However, in many cases this necessitates the protection of functional groups or requires other transformations like reduction or oxidation of functional groups after introduction of radiofluorine [14].

The indirect method involves build-up syntheses, that is, changing the carbon skeleton structure and starting from small molecules which themselves can be easily ^18^F-fluorinated by nucleophilic substitution. Such small ^18^F-labelled alkyl [15] or aryl [16] groups bear typically reactive functional groups for further transformation reactions. Those intermediates are used to synthesize more complex biological molecules which cannot be labelled with fluorine-18 due to mechanistic reasons or are not stable enough to tolerate direct ^18^F-fluorination conditions.

In the case of ^18^F-labelling of macromolecules like peptides, proteins, and antibodies, these small ^18^F-labelled intermediates are commonly called “prosthetic groups.” In the last decade progress has been made regarding the ^18^F-labelling of macromolecules [17, 18]. Besides the use of prosthetic groups several alternative methods have also been introduced, capable of using even mild and aqueous conditions, for example, chelated aluminum [19–21], boron- [22], and/or silicon-based [^18^F]fluoride acceptor groups [23–26]. The latter methods were also used for the synthesis of small molecules [27].

This review focuses on new developments regarding the use of small ^18^F-labelled intermediates for build-up syntheses of biologically active compounds. The ^18^F-labelling of macromolecules and the click chemistry approach are not considered. Those special topics of ^18^F-labelling can be found in other contributions to this issue [28–30].

## 2. ^**18**^F-Fluorinating Agents

As starting material for all chemical syntheses either aqueous [^18^F]fluoride or gaseous [^18^F]F_2_ is used, both of which are generally produced at a cyclotron via the ^18^O(p,n)^18^F nuclear reaction [31]. The nucleophilic [^18^F]fluoride ion is available in no-carrier-added (n.c.a.) form which allows the synthesis of radiotracers with high specific activity. In contrast, in-target produced [^18^F]F_2_ is available only in carrier-added (c.a.) form which leads to radiotracers with low specific activity.

Historically, for important radiopharmaceuticals like 2-[^18^F]fluoro-2-deoxy-D-glucose ([^18^F]FDG) and 6-[^18^F]fluoro-L-dopa only electrophilic ^18^F-fluorination was available. Today this method is rarely used because of the need of carrier for [^18^F]F_2_ production. Thus, the use of electrophilic ^18^F-fluorination is limited to nontoxic compounds as well as to those that can be applied with a low specific activity. Also, since [^18^F]F_2_ is very reactive and ^18^F-labelled side products are formed, less reactive electrophilic ^18^F-agents were developed [32]. More recently, the synthesis of N-[^18^F]fluorobenzenesulfonimide (NFSi) was described, which is a highly stable, reactive and selective electrophilic ^18^F-labelling agent and allows the synthesis of ^18^F-labelled allylic fluorides and *α*-fluorinated ketones from allylsilanes and silyl enol ethers, respectively [33].

**Figure 1:**
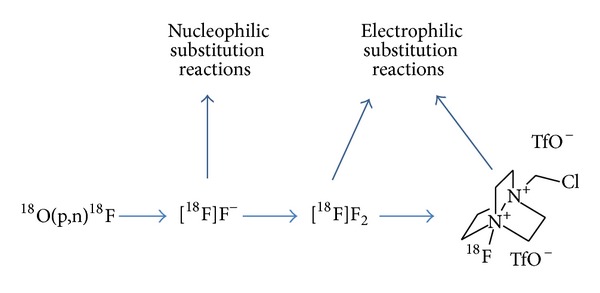
Nuclear reactions to produce fluorine-18 and the ^18^F-fluorinating agents [^18^F]fluoride, [^18^F]fluorine gas, and [^18^F]selectfluor bis(triflate).

An alternative method using a “posttarget” synthesis of [^18^F]F_2_ leads to moderate specific activity of up to 24.7 GBq/*μ*mol, starting from n.c.a. [^18^F]fluoride [34]. It was recently revisited for the radiosynthesis of [^18^F]selectfluor bis(triflate), the ^18^F-labelled form of (1-chloromethyl-4-fluorodiazonia-bicyclo[2.2.2]-octane bis-(tetrafluoroborate)), an easy to handle and stable electrophilic fluorinating reagent (cf.  Figure 1) [35]. This reagent could successfully be used for the silver(I)-mediated ^18^F-fluorination of electron-rich arylstannane models and intermediates, as well as for the preparation of 6-[^18^F]fluoro-L-DOPA [36], albeit all with limited specific activity of 3.7 ± 0.9 GBq/*μ*mol.

## 3. Aliphatic Intermediates

**Figure 2:**
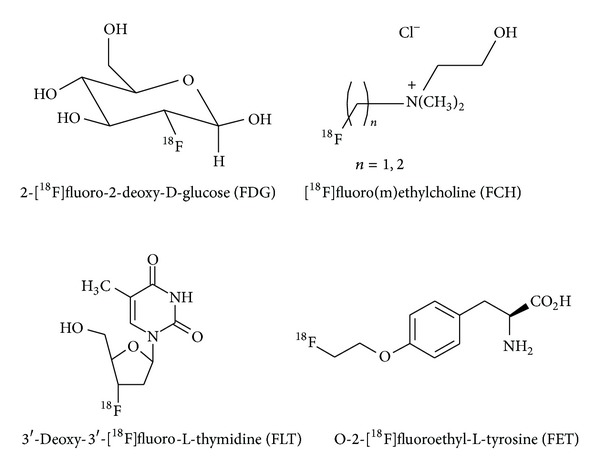
Important ^18^F-labelled radiotracers in clinical use.

Aliphatic ^18^F-fluorination is certainly the most prominent method for ^18^F-labelling [32], and important PET-radiotracers for clinical use are aliphatically ^18^F-labelled compounds which fulfill these requirements, for example, [^18^F]FDG, 3′-deoxy-3′-[^18^F]fluorothymidine ([^18^F]FLT), [^18^F]fluoro(m)ethylcholine, and O-2-[^18^F]fluoroethyl-L-tyrosine ([^18^F]FET) (cf.  Figure 2) [15, 37, 38]. [^18^F]Fluoro(m)ethylcholine is an example for ^18^F-labelled endogenous compounds, whereas [^18^F]FDG and [^18^F]FLT are ^18^F-labelled deoxy derivatives of the corresponding endogenous substances. In all cases a proton is replaced by a fluorine atom without changing the carbon skeleton of the original compound. In contrast, [^18^F]FET is an example of an endogenous ^18^F-labelled compound where the introduction of the radionuclide is performed by an ^18^F-fluoroalkylation reaction. Here, the ^18^F-label is introduced into the molecule by addition of further C-atoms which means that the skeleton of the molecule is significantly changed. Other examples of this kind of reaction are the ^18^F-fluoroacylation and ^18^F-fluoroamidation reactions which are widely used for labelling of macromolecules [39], most often in aqueous solution.

### 3.1. Intermediates for Nucleophilic Substitution and Other Coupling Reactions

**Figure 3:**
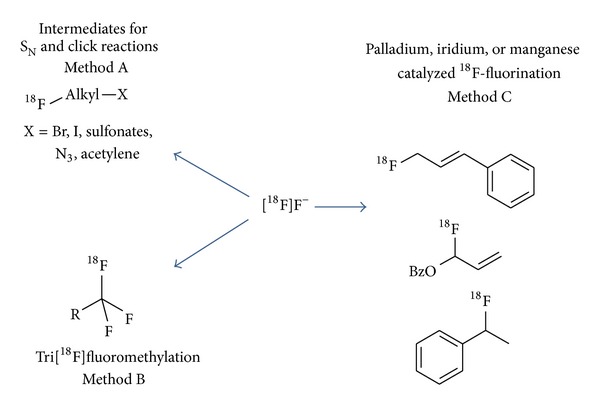
Pathways for aliphatic ^18^F-labelling intermediates starting from n.c.a. [^18^F]fluoride.

The synthesis of intermediates for ^18^F-fluoroalkylation is characterized by a two- or three-step procedure (cf.  Figure 3) [40]. First, [^18^F]fluoride is introduced into a molecule using precursors containing a good leaving group. The ^18^F-labelled precursor is then isolated and purified before coupling with a further molecule.

In the first step the [^18^F]fluoride has to be separated from the target water and activated for a nucleophilic substitution reaction. The standard conditions of these basic methods are described in several reviews [11, 32, 41]. A simplification of this approach was achieved by water removal on a strong anion-exchange resin [42] or by use of strong organic bases as additives replacing the inorganic bases or salts classically used in the resin eluent [43–46]. Instead of trapping on anion-exchange resins n.c.a. [^18^F]fluoride can also be separated by electrochemical methods which are useful to minimize the reaction volume especially for the use in microfluidic systems [47–51]. The use of mixtures of nonpolar tert-alcohols with acetonitrile as a reaction medium enhanced the reactivity of cesium[^18^F]fluoride or tetrabutylammonium [^18^F]fluoride and reduced the formation of typical by-products compared to those conventionally obtained only with dipolar aprotic solvents [52, 53].

Bromine and iodine and several sulfonate derivatives serve generally as leaving groups for a nucleophilic aliphatic radiofluorination [15, 40, 54, 55]. Alternatively, in the case of preparation of O-[^18^F]fluoromethylated aliphatic and aromatic ethers, the 1,2,3-triazolium triflate group serves as a very good nucleofuge for displacement by the [^18^F]fluoride ion [56].

The purification of ^18^F-fluorinating agents is performed by HPLC, solid phase extraction (SPE), or distillation. The main challenge is the complete separation of the ^18^F-labelled intermediate from the precursor which also would act as reaction partner in the following coupling step. This leads to unwanted side reactions which could lower the radiochemical yield (RCY) or necessitate a higher concentration of the precursor for the subsequent coupling reaction. A purification of the ^18^F-fluorinating agent via HPLC (or GC) is very effective and is often used [57–59], but it is more inconvenient for automatization [60, 61]. The use of SPE [62–64] or a distillation process for purification is principally easier to automate [40]. For instance, 1-bromo-3-(nitrobenzene-4-sulfonyloxy)-propane as starting precursor will be retained in the reaction vessel during the distillation process of 1-bromo-3-[^18^F]fluoropropane, due to its very high boiling point, thus eliminating the risk of formation of pseudocarrier [65]. In a few cases the direct coupling of the ^18^F-labelled intermediate was performed without former separation and purification [66].

Another possibility for simplified workup is the use of fluorous solid phase extraction (FSPE). A nucleophilic ^18^F-fluorination of fluorous-tagged precursors can easily be purified by FSPE regardless of the affinity of the untagged substrate for the stationary phase. FSPE-purified labelled compounds can then be used in subsequent reactions or more easily purified by HPLC before administration [67, 68]. A similar approach was performed using molecular imprinted polymers [69].

Coupling reactions of the ^18^F-fluorination agent with the desired target molecule are performed either by the use of a further leaving group, by the click chemistry approach [70], by Staudinger ligation [71–73], or by Pd(0) mediated reactions [74].

A series of arylsulfonates were prepared as nucleophile assisting leaving groups (NALG) in which the metal chelating unit is attached to the aryl ring by an ether linker. Under microwave irradiation and without the assistance of a cryptand, such as Kryptofix 2.2.2, primary substrates with selected NALGs led to a 2-3-fold improvement in radiofluorination yields over traditional leaving groups [75].

### 3.2. Tri[^18^F]fluoromethyl Group

The CF_3_ group has an electronegativity similar to that of oxygen [76] and is characterized by a large hydrophobic parameter as measured by the relative partition coefficient [77]. The trifluoromethyl group is an important pharmacophore present in many biologically active pharmaceutical and agrochemical drugs. The increased lipophilicity and a superior metabolic stability compared to that of the trifluoromethyl analogues often account for an improved activity profile [78]. Thus, radiolabelled trifluoromethyl groups are of potential interest to facilitate drug discovery. Earlier attempts to synthesize an ^18^F-labelled trifluoromethyl group were also characterized by low RCY and low specific activity due to decomposition of the target material [79–81].

The recently published developments can be divided in aliphatic and aromatic tri[^18^F]fluoromethylation reactions (cf.  Figure 3, method B).

**Figure 4:**
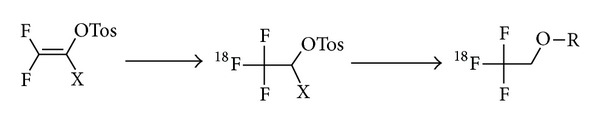
New aliphatic tri[^18^F]fluoromethylation.

A novel, one-step method for nucleophilic radiosynthesis of aliphatic tri[^18^F]fluoromethyl groups using the n.c.a. [^18^F]fluoride ion under relatively mild conditions was developed by incorporation of the radiolabel by an equivalent nucleophilic addition of H[^18^F]F to the 1-tosyl-2,2-difluorovinyl group (cf.  Figure 4). The tosylate function then serves as leaving group in a subsequent coupling step [82, 83]. The specific activity of the tri[^18^F]fluoromethylether was determined to be 86 MBq/nmol. The need of a double bond to achieve the addition of the [^18^F]fluoride limits this reaction to aliphatic tri[^18^F]fluoromethylations.

**Figure 5:**
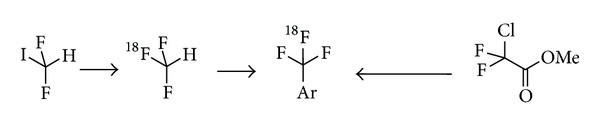
Aromatic tri[^18^F]fluoromethylation reactions.

Aromatic tri[^18^F]fluoromethyl groups were formerly synthesized using hardly accessible aromatic-CF_2_Br groups [84]. Two new approaches were published quite recently (cf.  Figure 5). Both methods start with an aliphatic precursor which is first labelled with fluorine-18 and then coupled to the benzene ring. In a two-step procedure tri[^18^F]fluoromethane ([^18^F]fluoroform) available from difluoroiodomethane and [^18^F]fluoride [85] is coupled in a copper(I) mediated reaction to aromatic halides using potassium tert-butoxide as base. The RCY was determined up to 65% with a specific activity of up to 50 GBq/*μ*mol [86]. This method has recently been improved performing a one-pot synthesis in the presence of copper(I)bromide, N,N-diisopropyl-N-ethylamine, and the corresponding iodoarene without separation of the [^18^F]fluoroform intermediate [87]. The RCYs of the desired tri[^18^F]fluoroarenes were determined with up to 90%, but no information on the specific activities was given.

An alternative method is used in a one-pot process. The trifluoromethylation agent [^18^F]CF_3_Cu, generated* in situ* from methyl chlorodifluoroacetate, CuI, TMEDA, and [^18^F]fluoride, is coupled to (hetero)aryl iodides in RCYs ranging from 17 to 87% [88]. A drawback of this procedure is still the relative low specific activity of 0.1 GBq/*μ*mol exemplified so far only for 4-tri[^18^F]fluoromethyl nitrobenzene. However, the method enables an efficient tri-[^18^F]fluoromethylation of complex molecules like [^18^F]fluoxetine. N-Boc protected [^18^F]fluoxetine was readily prepared in 37% RCY. The subsequent N-Boc deprotection delivered [^18^F]fluoxetine with 95% yield. A more detailed review on the scope and limitations of the radiosynthesis of tri[^18^F]fluoromethyl groups is provided as part of this special issue [89].

### 3.3. Palladium, Managnese and Iridium Catalyzed ^18^F-Fluorovinylation

Transition metal catalyzed allylic substitution is a powerful method for carbon–carbon and carbon–heteroatom bond formation (cf.  Figure 3 above, method C). These reactions encompass a wide variety of heteroatoms (N, O, and S) as nucleophiles [90]. In the field of ^18^F-chemistry a palladium catalyzed allylic fluorination reaction was developed and transferred to n.c.a. conditions yielding ^18^F-labelled cinnamyl fluoride starting from [^18^F]TBAF, cinnamyl methyl carbonate, [Pd (dba)_2_], and triphenylphosphine in anhydrous acetonitrile [91].

Further, a rapid allylic fluorination method utilizing trichloroacetimidates in conjunction with an iridium catalyst has been developed. The reaction is performed at room temperature without the need of inert gas atmosphere and relies on the Et_3_N*·*3HF reagent to provide branched allylic fluorides with complete regioselectivity. This high-yielding reaction can be carried out on a multigram scale and shows considerable functional group tolerance. The use of Kryptofix 2.2.2/K_2_CO_3_ allowed an incorporation of fluorine-18 within 10 min [92]. The RCY of allylic [^18^F]fluoride was determined to be 38%. A specific activity for the aforementioned reactions, however, was not reported.

A new method enables the facile n.c.a. ^18^F-labelling of aliphatic C–H bonds in benzylic position using manganese salen catalysts with RCY ranging from 20% to 72% within 10 min without the need for preactivation of the labelling precursor [93].

## 4. Aromatic and Heteroaromatic Intermediates

### 4.1. ^18^F-Labelled Aromatic and Heteroaromatic Intermediates by Classic Approaches

**Figure 6:**
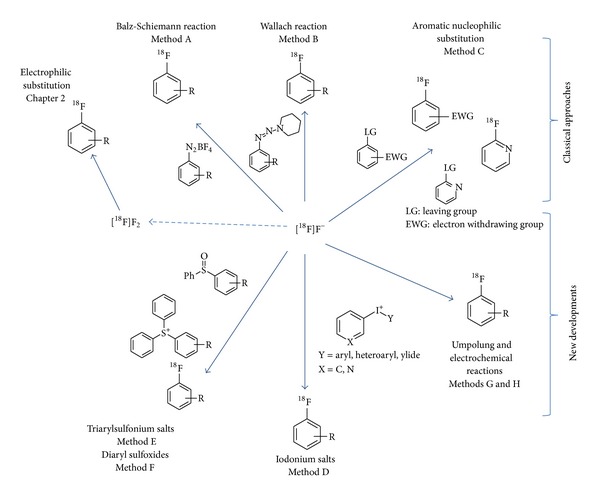
Pathways for aromatic ^18^F-labelling.

Historically, the use of the Balz-Schiemann or Wallach reaction was the first attempt to synthesize ^18^F-labelled aromatic rings starting from [^18^F]fluoride (cf.  Figure 6, method A) [94, 95]. However, the thermal decomposition of the corresponding aryl diazonium salts and of the aryl triazenes is characterized by low RCY, a low specific radioactivity, and extensive by-product formation [95]. The use of tetrachloroborate or 2,4,6-triisopropylbenzenesulfonate as counterions led to improvements of the Balz-Schiemann reaction which enables the synthesis of [^18^F]fluoroarenes in 39% RCY at the n.c.a. level, exemplified for 4-[^18^F]fluorotoluene [96]. In a recently published study these nucleophilic ^18^F-labelling methods were reinvestigated using polymer bound aryl diazonium salts and aryl triazenes [97]. The solid phase supported de-diazofluorination using arenediazonium cations, ionically bound to a sulfonate functionalised ion exchange resin, was, however, not suitable for nucleophilic ^18^F-labelling of aromatic compounds, whereas the solid supported triazene yielded the ^18^F-labelled product in a reasonable RCY of 16%.

**Figure 7:**
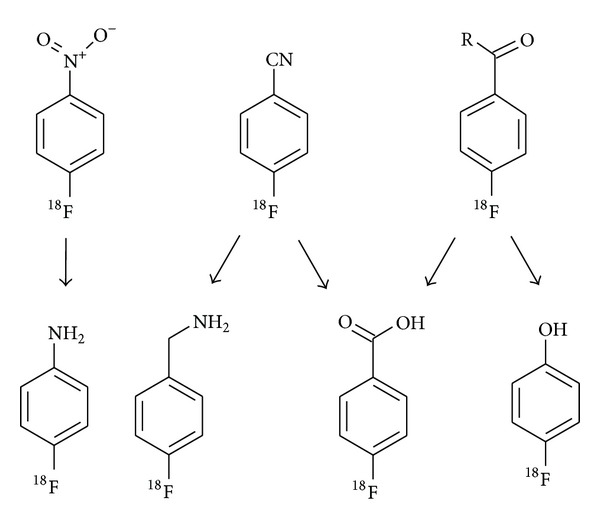
^18^F-labelled aromatic small molecules available by S_N_Ar reactions used as intermediates and further important ^18^F-intermediates derived therefrom.

Most successful for the introduction of fluorine-18 into aromatic rings is the conventional aromatic nucleophilic substitution (S_N_Ar) reaction using the [^18^F]fluoride anion to displace a suitable leaving group from an electron deficient benzene ring. As leaving groups serve halides, the nitro and the trimethylammonium function. The activation of the aromatic ring is usually achieved by suitable functional groups with a–M effect like the carbonyl, carboxyl, cyano, and nitro group [32]. These highly activating groups especially enable the efficient introduction of [^18^F]fluoride into aromatic rings to label small ^18^F-intermediates for build-up syntheses. The activating functionality is then converted by reduction, oxidation, or hydrolysis to nucleophilic groups for subsequent coupling reactions. The n.c.a. intermediates 4-[^18^F]fluoroaniline, 4-[^18^F]fluorobenzylamine [98, 99], 4-[^18^F]fluorobenzoic acid, or 4-[^18^F]fluorophenol (see Section 3.3), which are not directly achievable by a ^18^F-fluorination reaction, are obtained by these strategies (cf.  Figure 7) [16, 100]. 4-[^18^F]Fluorobenzaldehyde is also used in multicomponent reactions to yield ^18^F-radiotracers with the label positioned on an aryl moiety, not susceptible to direct nucleophilic fluorination [101].

**Figure 8:**
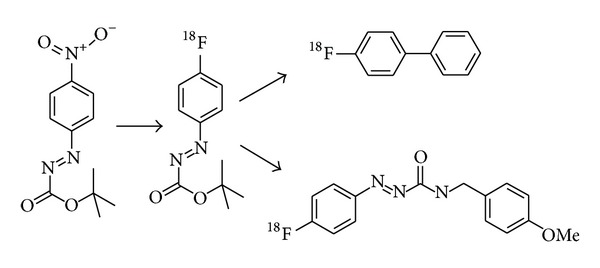
Radical arylation and substitution reactions of a ^18^F-labelled phenylazocarboxylic ester [102].

The azocarbonyl unit is a new group for activation of the arene ring by an S_N_Ar mechanism. The aromatic core of phenylazocarboxylic esters is highly activated towards nucleophilic aromatic ^18^F-substitution (cf.  Figure 8) [102]. This kind of compounds was converted in a radical arylation reaction into biaryl compounds or in substitutions at its carbonyl unit to produce azocarboxamides. Because of the high reactivity of the aryl radical, side products like [^18^F]fluorobenzene and 4-[^18^F]fluorophenol were also formed.

The conventional nucleophilic aromatic substitution reaction can principally be used for the n.c.a. ^18^F-labelling of aromatic rings in complex molecules [14]. However, the direct introduction of [^18^F]fluoride is often hampered by a lack of activation and further functional groups, especially those which have acidic protons. In the case of free amino, hydroxyl, or carboxylic acid functions the use of protecting groups is indispensable which have to be removed at the end of synthesis. Generally, the direct ^18^F-labelling of complex molecules enables the establishment of one-pot syntheses which is advantageous of being better introduced in a remote controlled synthesizer. In a multistep synthesis the intermediates have often to be purified (e.g., [103]) which hampers the installation in a synthesis module. Thus, one-pot syntheses are normally preferable over the build-up synthesis using several reactor vessels. There are exceptions to this rule, for example, when the build-up synthesis gives substantially higher RCYs [104].

In contrast to benzene, some heteroarenes like pyridine efficiently support the S_N_Ar reaction and can directly be used to prepare ^18^F-labelled heteroarenes in the 2- or 4-position [105–107]. Because of its straightforward feasibility, this method was even applied for radiofluorination of complex structures containing an azabenzoxazole [108], a 1,3-thiazole [109], a fluoropurine [110], a pyridine [111–118], a quinolinol [119], or a pyrimidine moiety [120].

### 4.2. New Developments on Radiofluorination of Arenes

In general, the examination of new methods for ^18^F-labelling of arene rings focuses on the late stage introduction of [^18^F]fluorine into complex organic molecules without the need of any transformation reaction afterwards. This principally simplifies the establishment of ^18^F-labelling methods in fully automated, remotely controlled synthesis units. However, these new methods are also useful for the synthesis of small intermediates for build-up synthesis. The novel methods of two prominent ones, [^18^F]fluorophenol and [^18^F]fluoro-halobenzene, are separately described (see Sections 4.3 and 4.4).

#### 4.2.1. Iodonium Salts (See Figure 6, Method D, and Figure 9)

The classical approach of n.c.a. nucleophilic aromatic ^18^F-substitution reactions is limited to activated arene rings. The use of diaryliodonium salts enables the introduction of n.c.a. [^18^F]fluoride into aromatic rings without further activation of strong electron withdrawing groups, which was first demonstrated in 1995 [121]. The reaction via an S_N_Ar mechanism leads to n.c.a. [^18^F]fluoroarenes and the corresponding iodoarenes. The nucleophilic attack on the diaryliodonium salt occurs preferably at the more electron-deficient ring and a steric influence of substituents, especially of ortho-substituted, could be observed [95]. Further studies have recently been performed to examine the possibilities and limitations of this reaction, with a focus on the synthesis of ortho- and meta-substituted arenes and the use of microreactors [122–124].

An interesting aspect here is that the reaction of diaryliodonium salts with [^18^F]fluoride is feasible in the presence of water, however, depending on the substituents present on the arene ring. Iodonium salts bearing a para- or ortho-electron-withdrawing group (e.g., p-CN) reacted rapidly (~3 min) to give the expected major [^18^F]fluoroarene product in useful, albeit moderate radiochemical yields even when the solvent had a water content of up to 28%. Iodonium salts bearing electron-withdrawing groups in metaposition (e.g., m-NO_2_) or an electron-donating substituent (p-OMe) gave low radiochemical yields under the same conditions. The finding that [^18^F]fluoroarenes, that having an ortho-alkyl substituent or an ortho- or a para-electron withdrawing group, can be synthesized without the need to remove irradiated water or to add a cryptand, could be attractive in some radiotracer production settings, particularly as this method saves time, avoids any need for automated drying of cyclotron-produced [^18^F]fluoride, and also avoids substantial loss of radioactivity through adsorption onto hardware surfaces [125].

In order to control the attack of the [^18^F]fluoride ion on the diaryliodonium salts it is important that one arene ring be more electron-rich than the ring to be labelled with fluorine-18. Here, the use of symmetrically substituted diaryliodonium salts [126] or the use of aryl(heteroaryl) iodonium salts [127] is an alternative to direct the ^18^F-labelling to the desired ring. More recently, the use of aryiodonium ylides became of interest for this purpose. The electron-rich status of the ylides, made, for example, from dimedone (5,5-dimethylcyclohexane-1,3-dione), even enables the synthesis of electron-rich arenes in high RCY [128]. This type of precursor has recently been demonstrated to be even suitable for complex molecules [129].

Some special intermediates like azide-containing diaryliodonium salts bearing an azidomethyl group on one aryl ring and with a 4-methoxy group on the second one enable the synthesis of click-labelling synthons up to 50 % RCY, even in the presence of a high fraction of water in the reaction solvent [130].

Halopyridinyl-(4′-methoxyphenyl)iodonium tosylates were used to rapidly produce [^18^F]fluorohalopyridines and in useful RCYs, including the otherwise difficult to access 3-[^18^F]fluorohaloisomers [131].

#### 4.2.2. Sulfur Activated Systems (See Figure 6 and Methods E and F)

Another newer method for the ^18^F-labelling of nonactivated aromatic compounds makes use of triarylsulfonium salts. The method is applicable to a wide range of substituted aryl systems including amides [132].

A new radiosynthetic method for producing n.c.a. [^18^F]fluoroarenes is based on the reactions of diaryl sulfoxides bearing electron-withdrawing paragroups with the [^18^F]fluoride ion. These reactions are relatively mild, rapid, and efficient. However, this reaction is limited to aromatic rings bearing an electron withdrawing function like the nitro, cyano, or trifluoromethyl group [133].

#### 4.2.3. Umpolungs Reactions (See Figure 6 and Methods G and H)

New concepts to synthesize ^18^F-labelled aromatic rings try to achieve fluoride-derived electrophilic n.c.a. fluorination reagents by fluoride umpolung [134, 135]. A preliminary realization of this concept was achieved by using a n.c.a. [^18^F]fluoride capture by a Pd(IV) complex to form an electrophilic ^18^F-fluorination reagent followed by a subsequent n.c.a. ^18^F-fluorination of palladium aryl complexes [136, 137]. Another kind of palladium catalyzed fluoride activation enables the synthesis of ^18^F-labelled 1-[^18^F]fluoronaphthalene in 33% RCY but only in the presence of fluoride carrier [138]. Another advanced method for a transition metal catalyzed late-stage radiofluorination relies on a one-step oxidative ^18^F-fluorination using a nickel aryl complex and a strong oxidation agent [139].

[^18^F]Fluoride can also be introduced into organic molecules by electrochemical oxidative fluorination via an aryl cation that undergoes rearomatization by loss of a proton. Oxidation of benzene in an electrolysis cell, using Et_3_N*·*3HF and Et_3_N*·*HCl in acetonitrile as the electrolyte, gave c.a. [^18^F]fluorobenzene in 17% RCY [140] and [^18^F]fluorophenylalanine in 10.5% RCY with a specific activity of 1.2 GBq/mmol [141].

However, the aim of all these methods is the late stage ^18^F-fluorination of electron-neutral and electron-rich aromatic compounds to simplify the synthesis of radiotracers. Regarding the palladium and nickel reactions, the precursor synthesis is often complex, has to be carefully handled under inert atmosphere, and needs high synthetic experience. This method is far from ideal, given the many reagents and demanding reaction conditions necessary, which hamper to fulfill a “good manufacturing practice” (GMP) pharmaceutical production [142, 143]. Thus, although principally applicable, their limitation and complexity do not warrant usefulness for the syntheses of build-up intermediates, as there are more efficient methods available for those molecules.

### 4.3. N.c.a. 4-[^18^F]Fluorophenol

4-[^18^F]Fluorophenol is a versatile structural unit for the synthesis of more complex radiopharmaceuticals bearing a 4-[^18^F]fluorophenoxy moiety. Former syntheses of n.c.a. 4-[^18^F]fluorophenol were made either by a modified Balz-Schiemann reaction or by hydrolysis of a 4-[^18^F]fluorobenzene diazonium salt with radiochemical yields of only 10–15% and 15–33% within 35 and 60 min, respectively [144]. These methods required either the preparation of an anhydrous tetrachloroborate or a two-step synthesis from [^18^F]fluoroaniline and were not established for radiotracer production.

A more reliable preparation of n.c.a. 2- and 4-[^18^F]fluorophenol was achieved using the Baeyer-Villiger reaction on ^18^F-labelled benzaldehyde, acetophenone, or benzophenone derivatives. Total radiochemical yields of about 25% were received using* m*-chloroperbenzoic acid as oxidant in the presence of trifluoroacetic acid [145]. The Baeyer-Villiger reaction of ^18^F-labelled benzophenone derivatives containing further electron withdrawing groups yielded up to 65% of 4-[^18^F]fluorophenol within 60 min with a high radiochemical purity. However, a considerable disadvantage of this method is the somewhat cumbersome work-up of the aqueous reaction mixture in order to isolate the product for its further use [146]. The formation of ^18^F-labelled 4-phenol derivatives by Baeyer-Villiger oxidation was, for example, applied to the direct ^18^F-fluorination of 6-[^18^F]fluoro-L-dopa [147].

**Figure 10:**
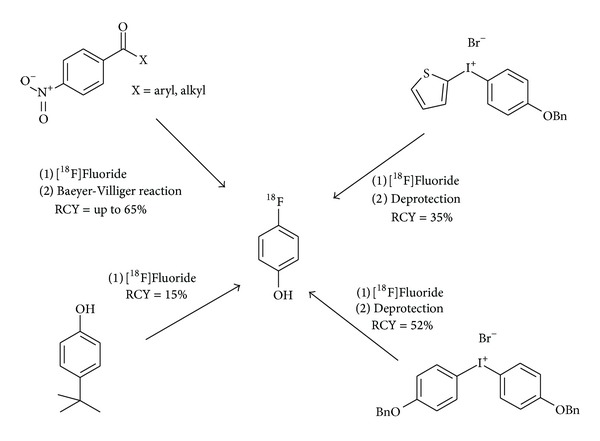
Methods for the synthesis of n.c.a. 4-[^18^F]fluorophenol.

A novel radiochemical transformation by an oxidative ^18^F-fluorination of tert-butylphenols uses the concept of an aryl umpolung (cf.  Figure 10) and is also applicable to other O-unprotected phenols. The reaction is performed at room temperature by applying a one-pot protocol and can also be performed in a commercially available microfluidic device [148].

Furthermore, aryl(thienyl) iodonium salts [149] and bis(4-benzyloxyphenyl) iodonium salts [150] have successfully been employed for the preparation of [^18^F]fluorophenol in a two-step procedure. Compared with the Baeyer Villiger method using benzophenone derivatives, this pathway saves 20 to 45 min of preparation time and delivers [^18^F]fluorophenol in an organic solution. So these methods are more useful for subsequent coupling reactions under anhydrous conditions. In contrast to the aryl umpolung reaction, the iodonium strategy, however, necessitates a deprotection step after the ^18^F-exchange.

### 4.4. N.c.a. 4-[^18^F]Fluorohalobenzene

**Figure 11:**
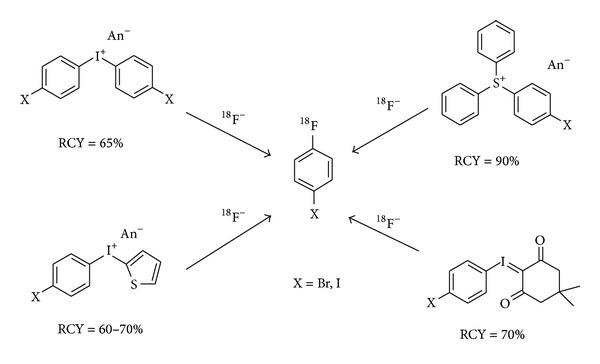
Most efficient one-step approaches for the n.c.a synthesis of [^18^F]fluoro-halobenzenes.

Recently, the synthesis of 4-[^18^F]fluorohalobenzenes has comprehensively been described [151]; here a few further aspects are added. 1-Bromo-4-[^18^F]fluorobenzene or 4-[^18^F]fluoro-1-iodobenzene serves as intermediates for C–C coupling reactions using Grignard-, lithium- [152], or palladium-mediated reactions [151, 153]. In Figure 11 the most efficient routes for the synthesis of n.c.a. 4-[^18^F]fluorohalobenzenes are illustrated. The use of symmetrically substituted diaryliodonium salts enables an efficient one-step synthesis of n.c.a. 1-bromo-4-[^18^F]fluorobenzene [154] as well as n.c.a. 4-[^18^F]fluoro-1-iodobenzene [155]. For the latter, the precursor synthesis is more challenging and has recently been improved [156]. The precursor syntheses of iodophenylthienyliodonium bromide and 4-iodophenyliodonium-(5-[2,2-dimethyl-1,3-dioxane-4,6-dione]) ylide [157] are easier to perform and the latter gave up to 70% RCY of 4-[^18^F]fluoro-1-iodobenzene [158]. The most efficient method for the one-step synthesis of 4-[^18^F]fluoro-1-iodobenzene is, however, the use of triarylsulfonium salts [132, 159] which leads to 90% RCY. A challenge, when using iodonium salts as precursor for the synthesis of 4-[^18^F]fluorohalobenzenes, is the formation of other nonradioactive halobenzene derivatives which are normally not separated from the ^18^F-labelled product and thus could hamper the final product separation.

## 5. Conclusion

The lack of universally useful methods for direct n.c.a. radiofluorination of complex molecules causes the wide use of ^18^F-labelled intermediates for the build-up synthesis of radiotracers. Nevertheless, multistep build-up syntheses of ^18^F-labelled radiotracers are confronted with several fundamental challenges, which often hamper a remotely controlled, large scale production by this type of reactions. Time consuming separation steps and the use of moisture or even air sensitive reagents complicate the automation of these build-up syntheses. Their application is therefore limited to specialized laboratories with the suitable equipment and experienced staff. The use of build-up reactions then often enables the only way to achieve the synthesis of new radiotracers. Once proven that a radiotracer has the potential to be a useful radiopharmaceutical for molecular imaging, most often ways can be found to establish its routine production via an alternative, simpler synthetic concept and/or by optimisation. Here, the novel developments in umpolung reactions or the improvements in iodonium chemistry in ^18^F-labelling of arenes are promising methods, which might also be effective for the late-stage ^18^F-fluorination of complex precursors. However, their suitability for daily routine GMP-production of radiopharmaceuticals remains to be elucidated.

## Figures and Tables

**Figure 9:**
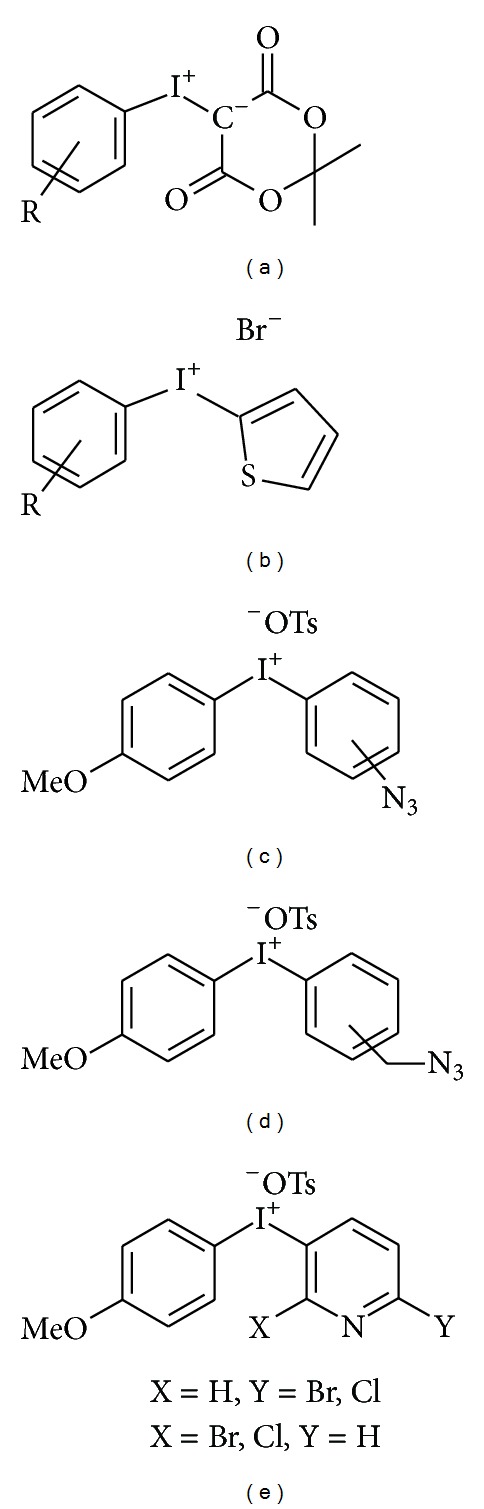
Structures of several iodonium salts for ^18^F-labelling: (a) iodonium ylide, (b) aryl(thienyl) iodonium salt, (c) [(azidomethyl)phenyl](4′-methoxyphenyl) iodonium tosylate, (d) [(azido)phenyl](4′-methoxyphenyl) iodonium tosylates, and (e) halopyridinyl-(4′-methoxyphenyl)iodonium tosylate.
